# Depleting RhoA/Stress Fiber-Organized Fibronectin Matrices on Tumor Cells Non-Autonomously Aggravates Fibroblast-Driven Tumor Cell Growth

**DOI:** 10.3390/ijms21218272

**Published:** 2020-11-04

**Authors:** Li-Tzu Huang, Chen-Lung Tsai, Shin-Huei Huang, Ming-Min Chang, Wen-Tsan Chang, Li-Hsin Cheng, Hung-Chi Cheng

**Affiliations:** 1 The Institute of Basic Medical Sciences, College of Medicine, National Cheng Kung University, 1 University Road, Tainan 70101, Taiwan; S16074144@gs.ncku.edu.tw (L.-T.H.); wtchang@mail.ncku.edu.tw (W.-T.C.); Lindsie0419@gmail.com (L.-H.C.); 2 Department of Biochemistry and Molecular Biology, College of Medicine, National Cheng Kung University, 1 University Road, Tainan 70101, Taiwan; jason830609@gmail.com (C.-L.T.); Shinhueihuang@outlook.com (S.-H.H.); 3 The Institute of Clinical Pharmacy and Pharmaceutical Science, College of Medicine, National Cheng Kung University, 1 University Road, Tainan 70101, Taiwan; hanxin.mmc@gmail.com

**Keywords:** Fibronectin (FN), pericellular FN matrices, actin stress fiber cytoskeleton, RhoA, tumor microenvironments, cancer associated fibroblasts, autonomous/non-autonomous regulation, in vitro tumor cell proliferation, in vivo tumor growth, cancer metastasis

## Abstract

Fibronectin (FN) expressed by tumor cells has been known to be tumor suppressive but the pericellular FN (periFN) assembled on circulating tumor cells appears to evidently promote distant metastasis. Whereas the regulation of periFN assembly in suspended cells has currently been under investigation, how it is regulated in adherent tumor cells and the role of periFN in primary tumor growth remain elusive. Techniques of RNAi, plasmid transfections, immunoblotting, fluorescence/immunohistochemistry staining, cell proliferation assays, and primary tumor growth in C57BL6 mice and Fischer 344 rats were employed in this study. We found that endogenously synthesized FN in adherent tumor cells was required for periFN assembly which was aligned by RhoA-organized actin stress fiber (SF). Depleting periFN on adherent tumor cells congruently promoted in vivo tumor growth but surprisingly did not autonomously impact on in vitro tumor cell proliferation and apoptosis, suggestive of a non-autonomous role of periFN in in vivo tumor growth. We showed that the proliferative ability of shFN-expressing tumor cells was higher than shScramble cells did in the presence of fibroblasts. Altogether, these results suggested that depriving RhoA/SF-regulated periFN matrices non-autonomously promotes fibroblast-mediated tumor cell growth.

## 1. Introduction

Metastasis is responsible for major cancer death [1,2]. Tumor cells initiate from normal, often epithelial, cells, the apoptotic and proliferative activities of which are dysregulated through oncogenic activation or inactivation by tumor suppressors and progress all the way from primary tissues to distant organs where they establish metastatic growth, which are autonomously and non-autonomously regulated in a temporal and spatial manner [1,3]. Cytoskeletons, particularly actin stress fibers (SFs), in tumor cells have often been found to critically contribute to tumorigenic and malignant cellular activities throughout the entire process of tumor progression [4,5,6,7]; In addition, tumor microenvironments (TMEs) including fibroblasts are essential in fostering tumor cells in every step of tumor progression [8].

Fibronectin (FN), a multifunctional extracellular matrix glycoprotein, distinctly participates in tumor progression in forms of polymers [1]. It has recently been reported that pericellular FN (periFN) assembled on circulating cell surfaces promotes tumor colonization, extravasation, and metastatic growth in lungs [9,10,11,12,13]. Indeed, accumulating experimental and clinical evidence indicates positive roles of FN in cancer metastasis, poor prognosis, and increased mortality [14,15,16,17,18]. However, FN expression in primary tumor tissues has been controversial. For example, abundant evidence shows that matrix-depositing FN polymers to which tumor cells adhere promote their proliferative activities [19,20,21,22]. Conversely, numerous reports have demonstrated that silencing or decreasing endogenous FN expression greatly enhances tumor growth [23,24,25,26,27], implicating that FN synthesized by tumor cells plays an inhibitory role in tumor cell proliferation. Like in suspended tumor cells [9,11,12], the endogenously synthesized FN in adherent tumor cells often presents itself as polymeric periFN matrices, a shared characteristic with stromal cells like fibroblasts, epithelial cells, and endothelial cells [28,29]. Although the regulations of periFN assembled on adherent normal cells [30,31,32,33] and suspended tumor cells [11,12,13] have been explored, how it is regulated on adherent tumor cells and whether it is autonomously involved in tumor proliferation and in vivo tumor growth are, nevertheless, less clear.

Extensive studies indicate that the periFN assembly on normal adherent cells is regulated by the small GTPase RhoA-organized SF cytoskeleton [28,29,32,33]. However, less has been explored as to the regulation of periFN matrix assembly by RhoA/stress fiber on adherent tumor cells. It has been reported that renal cancer cells deficient in von Hippel–Lindau (VHL) protein lack the ability to assemble periFN matrices, implicating that periFN displays a role in suppressing tumorigenesis [34,35,36]. Inadequate RhoA activity has been shown to be responsible for the lack of periFN assembly in VHL^−/−^ renal cancer cells [37]. Whether periFN assembly is regulated by RhoA activation in other types of VHL^+/+^ cancer cells remains to be demonstrated. Interestingly, VHL protein has been shown to promote actin SF assembly [38]. Altogether, these findings lead to a possibility that periFN assembly on tumor cells is organized and aligned by SF cytoskeleton that is regulated by activated RhoA.

That TMEs closely participate in tumor initiation and progression has been recognized over the past decades [39,40]. While microenvironments surrounding early-stage tumors play an anti-tumor role, the tolerated and survived cancer cells subsequently become competent in driving the TMEs into pro-tumor milieus [41]. TMEs are composed of specifically organized extracellular matrices and a multicellular system, including mesenchymal, endothelial, and hematopoietic origins, which intimately interact with tumor cells, contributing to tumorigenesis [39]. In TMEs, fibroblasts are among stromal cells one of the most influential mesenchymal components, providing not only physical support but also chemical signaling for promoting tumor growth [19]. In addition to the likelihood of autonomous regulation of periFN on tumor growth, whether periFN assembled on tumor cells exerts an effect on fibroblasts which, in turn, non-autonomously regulate tumor cell proliferation is intriguing to be explored.

Here, we found that cancerous FN synthesis was required for the periFN assembly on tumor cells. The FN matrices were well aligned with and regulated by SF cytoskeletons in a RhoA activity-dependent manner. Conversely, periFN assembly was dispensable to SF organization. In contrast to its anti-metastatic functionality, deprivation of periFN on tumor cells significantly promoted in vivo primary tumor growth in the subcutaneously inoculated position, implicative of a suppressive role of periFN in tumor cell proliferation. Surprisingly, neither proliferative nor apoptotic activities were affected in periFN-deprived tumor cells, suggestive of the involvement of TMEs in such pro-tumorigenic effect of periFN deprivation. Indeed, tumor cell proliferation was significantly promoted once periFN-depleted tumor cells were co-cultured with fibroblasts. Altogether, our results supported that depletion of stress fiber-organized fibronectin matrix does not alter tumor proliferative activity but aggravates in vivo tumor growth.

## 2. Results

### 2.1. The Endogenous FN-Dependent periFN Assembly on Tumor Cells Is Regulated by SF Actin Cytoskeleton

**Figure 1:**
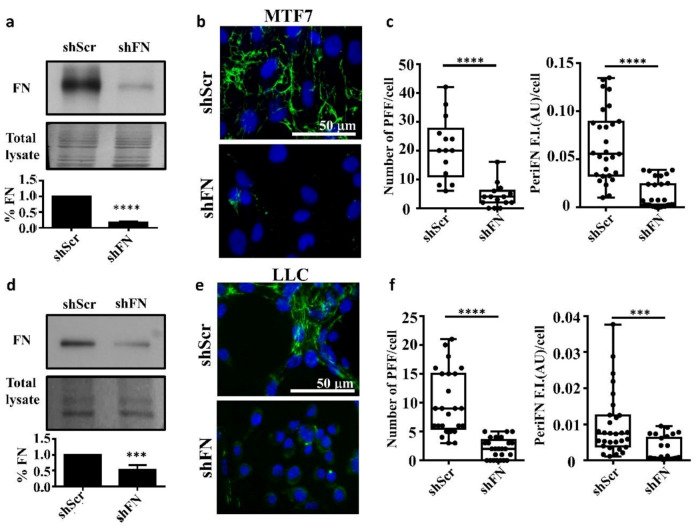
Endogenously synthesized fibronectin (FN) is required for pericellular FN (periFN) matrix assembly of tumor cells. (**a**) Upper panel: IB assays for the FN expression in shScr- or shFN-expressing MTF7 cells. Middle panel: Total lysate was the Coomassie blue staining of the same blotted membrane serving as an equal loading control for FN IB in the upper panel. Lower panel: Densitometry quantification of the FN IB relative to total lysate and FN in shFN cells relative to that in shScr cells with Image J. (**b**) IF staining for periFN (green) assembled on shScr- or shFN-expressing MTF7 cells. DAPI dye (blue) was used for nuclear staining. Note: both images share the same scale bar in the upper panel. (**c**) Quantification of the IF staining for periFN in (**b**) with Image J. PFF: periFN filament with length longer than 7 µm was counted as one filament; F.I.: fluorescence intensity of total PFFs; AU: arbitrary unit. (**d**) The same IB assay, total lysate, and densitometry quantification for LLC cells as those for MTF7 cells in (**a**). (**e**) The same IF staining for LLC cells as that for MTF7 cells in (**b**). Note: both images share the same scale bar in the upper panel. (**f**) The same densitometry quantification of the IF staining for LLC cells as that for MTF7 cells in (**c**). Note: all experiments were repeated at least three times. ***: *p* < 0.001; and ****: *p* < 0.0001.

To test whether endogenously expressed FN is required for the periFN assembly on tumor cells in adherent status, we stably silenced FN expression in adherent rat mammary adenocarcinoma MTF7 cells and mouse Lewis lung cancer LLC cells and examined their FN expressions (Figure 1a for MTF7 cells and Figure 1d for LLC cells) and surface periFN matrices (Figure 1b,c for MTF7 cells and Figure 1e,f for LLC cells). We quantitatively found that endogenous FN expressions and the levels of periFN assembly were significantly higher in the control shScr-MTF7 cells (Figure 1a (lower panel) and Figure 1c, respectively) and shScr-LLC cells (Figure 1d (lower panel) and Figure 1f, respectively) than in shFN-silenced MTF7 and LLC cells.

**Figure 2:**
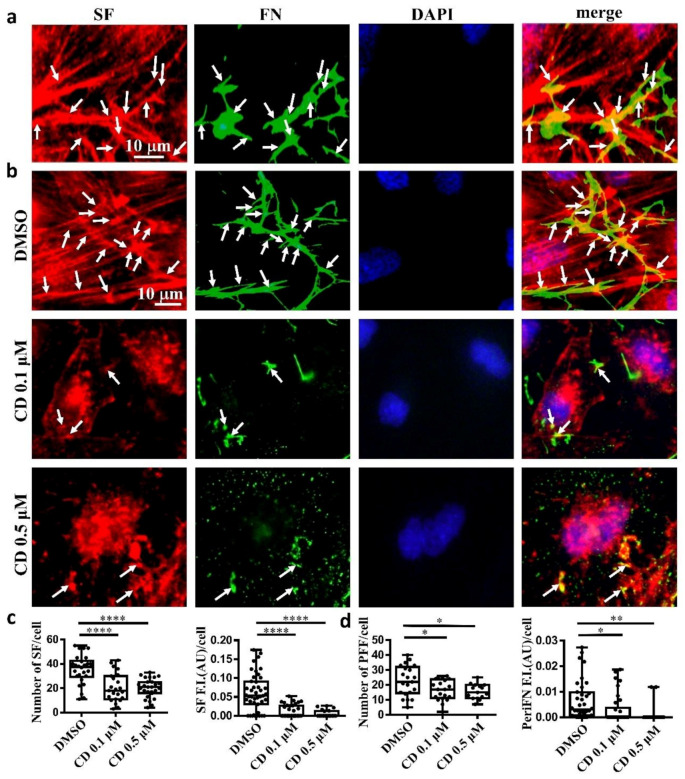
Tumor FN matrix is aligned with and regulated by stress fiber (SF) cytoskeleton. (**a**) MTF7 cells were double-stained with Alexa 594-conjugated phalloidin for actin filaments (in red) and anti-FN pAb for periFN (in green). DAPI dye (blue) was used for nuclear staining. Note: the scale bar denoted in the image of left panel is applied to the other three images. (**b**) DMSO- or 0.1/0.5 µM cytochalasin D (CD)-treated MTF7 cells were double-stained with Alexa 594-conjugated phalloidin (in red) and anti-FN pAb (in green). Note: the scale bar denoted in the image of upper left panel is applied to the other 11 images. (**c**) Quantification of the SF filaments with length longer than 13 µm in (**b**) with Image J. (**d**) Quantification of the periFN in (**b**) with Image J. Arrows indicate colocalizations between SF and periFN filaments. Note: all experiments were repeated at least three times. *: *p* < 0.05; **: *p* < 0.01; and ****: *p* < 0.0001.

Next, we asked whether, like on normal cells, periFN assembled on adherent tumor cells is colocalized with SF cytoskeleton. We indeed found that the filamentous periFN matrices on MTF7 cells were well colocalized with intracellular SF cytoskeleton (Figure 2a).

**Figure 3:**
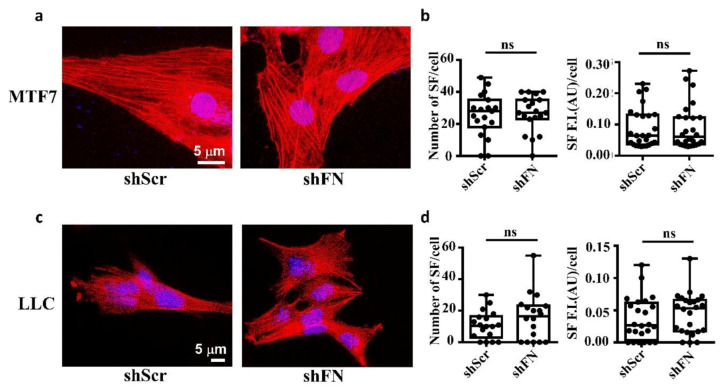
Deprivation of tumor periFN does not alter stress fiber organization. (**a**) Actin SF staining with Alexa 594-conjugated phalloidin in shScr or shFN-expressing MTF7 cells. Nuclei stained with DAPI are in blue. Note: Both images share the same scale bar in the left panel. (**b**) Quantification of the actin filaments in (**a**). (**c**) Actin SF staining with phalloidin in shScr or shFN-expressing LLC cells. Note: Both images share the same scale bar in the left panel. (**d**) Quantification of the SF filaments in (**c**). Note: all experiments were repeated at least three times. Ns: no significance.

Consistently, we clearly demonstrated that treating MTF7 cells with various concentrations of cytochalasin D, a potent inhibitor of actin polymerization, dose-dependently depolymerized SF cytoskeleton (Figure 2b (left panels) and Figure 2c) and suppressed the assembly of filamentous periFN matrices (Figure 2b (panels next to the left panels) and Figure 2d). Nevertheless, silencing endogenous FN expression did not affect SF actin cytoskeleton organization in MTF7 cells (Figure 3a,b) and LLC cells (Figure 3c,d). Altogether, these results suggested that the periFN matrix assembly on adherent tumor cells requires endogenously expressed FN and is regulated and aligned by polymerized SF cytoskeleton and periFN assembly is dispensable to SF formation.

### 2.2. The Stress Fiber-Organized Tumor periFN Assembly Is Regulated by RhoA Activity

**Figure 4:**
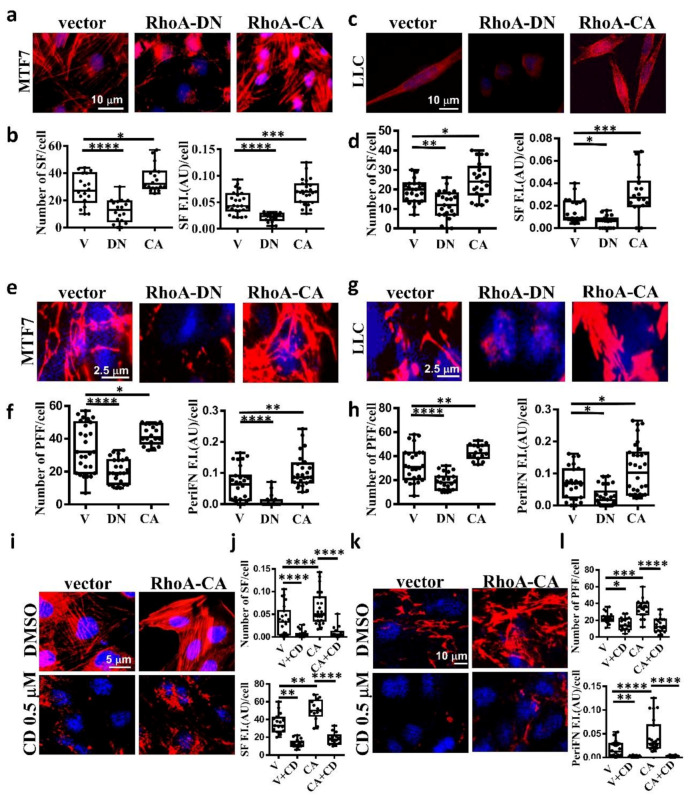
SF cytoskeleton mediates RhoA-activated tumor periFN assembly. (**a**) actin SF staining with Alexa 594-conjugated phalloidin for MTF7 cells ectopically expressing vector alone, RhoA-DN, or RhoA-CA. (**b**) Quantification of SFs in (a) with Image J. (**c**) and (**d**) The same actin SF staining with Alexa 594-conjugated phalloidin and quantification for LLC cells as those for MTF7 cells in (**a**) and (**b**), respectively. (**e**) PeriFN IF staining for MTF7 cells ectopically expressing vector alone, RhoA-DN, or RhoA-CA. (**f**) Quantification of periFN filaments in (**e**) with Image J. (**g**) and (**h**) The same PeriFN IF staining and quantification for LLC cells as those for MTF7 cells in (**e**) and (**f**), respectively. (**i**) The same actin SF staining with Alexa 594-conjugated phalloidin for DMSO- or CD (0.1 µM)-treated MTF7 cells ectopically expressing vector alone or RhoA-CA as those in (**a**). (**j**) Quantification of SFs in (**i**) with Image J. (**k**) The same periFN IF staining for DMSO- or CD (0.1 µM)-treated MTF7 cells ectopically expressing vector alone or RhoA-CA as those in (**i**). (**l**) Quantification of periFN filaments in (k) with Image J. Note: all images in (**a**), (**c**), (**e**), (**g**), (**i**), and (**k**) share the same scale bars respectively depicting the magnitude of images for tumor cells expressing vector and all experiments were repeated at least three times. *: *p* < 0.05; **: *p* < 0.01; ***: *p* < 0.001; and ****: *p* < 0.0001.

It has been well evidenced in fibroblasts that SF actin cytoskeleton is specifically regulated by small G protein RhoA [32,42]. To explore whether SF is also regulated by RhoA in tumor cells, we transfected MTF7 and LLC cells with vector alone, dominant-negative Thr19Asn RhoA mutant (RhoA-DN), which loses binding ability to guanidine nucleotides but is able to compete with endogenous GDP-bound RhoA for guanidine nucleotide exchanging factor (GEF)-binding and prevents GDP bound on endogenous RhoA from being exchanged into GTP, and constitutively active Gln63Leu RhoA mutant (RhoA-CA), in which the bound GTP can no longer be hydrolyzed into GDP [43,44]. We found that the SF assembly was significantly reduced on RhoA-DN-transfected MTF7 cells (Figure 4a in left and middle panels and Figure 4b for the corresponding quantifications) and LLC cells (Figure 4c in left and middle panels and Figure 4d for the corresponding quantifications). On the other hand, the SF formation appeared to be markedly enhanced on RhoA-CA-transfected MTF cells (Figure 4a in left and right panels and Figure 4b for the corresponding quantifications) and LLC cells (Figure 4c in left and right panels and Figure 4d for the corresponding quantifications). These results suggested that SF formation in adherent tumor cells is positively regulated by RhoA activity. We next examined periFN assemblies on these tumor cells. In line with the effects on SF actin cytoskeleton, periFN was dramatically reduced in the RhoA-DN-transfected MTF7 cells (Figure 4e in left and middle panels and Figure 4f for the corresponding quantifications) or LLC cells (Figure 4g in left and middle panels and Figure 4h for the corresponding quantifications) but enhanced in the RhoA-CA-transfected MTF cells (Figure 4e in left and right panels and Figure 4f for the corresponding quantifications) or LLC cells (Figure 4g in left and right panels and Figure 4h for the corresponding quantifications). Treating the RhoA-CA-transfected MTF7 cells with cytochalasin D completely disrupted their SF actin filaments (Figure 4i,j) and periFN assembly (Figure 4k,l), suggesting that SF actin cytoskeleton mediates the RhoA-activated periFN matrix assembly on adherent tumor cells.

### 2.3. PeriFN Assembled on Tumor Cells Displays A Suppressive Role in In Vivo Tumor Growth

**Figure 5:**
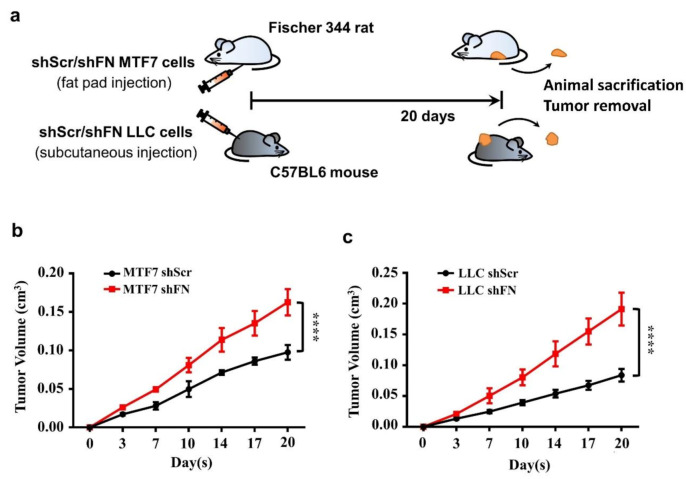
Alteration of periFN assembly with shFN impacts in vivo tumor growth. (**a**) Schematic illustration for in vivo MTF7 or LLC tumor growth animal models. Volumes of shScr- or shFN-expressing MTF7 (**b**) or LLC (**c**) tumor masses grown in mammary fat pads of Fischer 344 rats or subcutaneous tissues of C57BL6 mice, respectively, were measured. Note: in vivo tumor growth experiments were performed in five mice or rats per group. ****: *p <* 0.0001.

Cancerous FN expression has been deemed to be tumor suppressive [23,24,25,26]. To test such observations in in vivo tumor growth, we subcutaneously inoculated shScr- or shFN-LLC cells in C57BL6 mice and shScr- or shFN-MTF7 cells in Fischer 344. We showed that mice bearing shFN-MTF7 or -LLC cells carried larger tumor sizes than those bearing shScr-MTF7 or -LLC cells did (Figure 5b,c, respectively), suggesting that periFN matrix assembly indeed plays a suppressive role in in vivo tumor growth.

### 2.4. Demolishing periFN Does not Promote Tumor Cell Proliferative Activity

**Figure 6:**
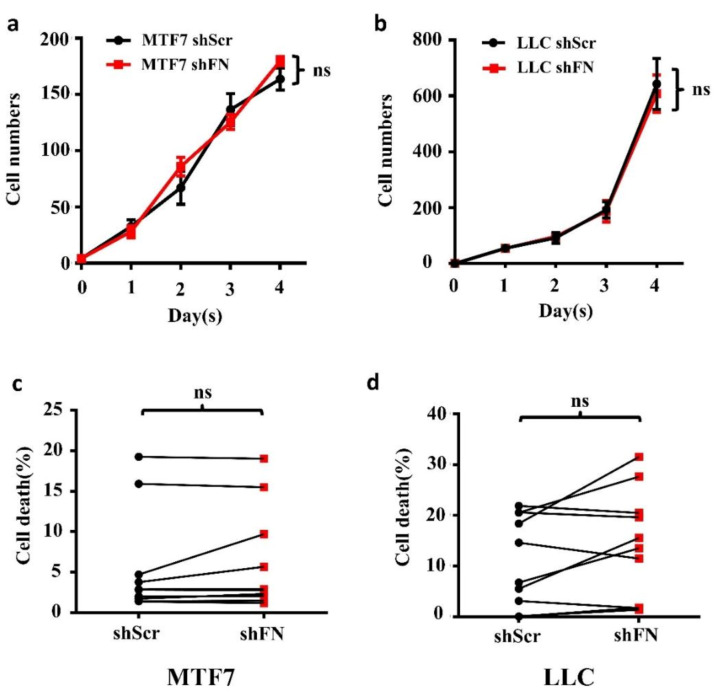
Depletion of tumor periFN does not affect tumor cell proliferation nor apoptosis. Cell proliferation assays (**a**,**b**) and cell apoptosis measurements with Annexin V/PI double staining as presented by a before–after graph (**c**,**d**) were performed in shScr- or shFN-expressing MTF7 (**a**,**c**) or LLC (**b**,**d**) cells. Note: all experiments were repeated at least three times. Ns: no significance.

To examine whether the enhanced in vivo tumor growth by depletion of periFN matrices on tumor cells was autonomously and directly caused by elevated tumor cell proliferation, we performed real-time cell proliferation assays. Surprisingly, neither did we observe any difference in in vitro tumor cell proliferation between the two cell lines (Figure 6a,b), nor did we detect any decrease of cell apoptosis (Figure 6c,d). These results suggested that cancerous FN expression and periFN assembly do not participate in autonomous tumor suppression but, importantly, suppress in vivo tumor growth in a non-autonomous manner.

### 2.5. Depleting Cancerous PeriFN with shFN Non-autonomously Promotes Fibroblast-Mediated Tumor Growth

**Figure 7:**
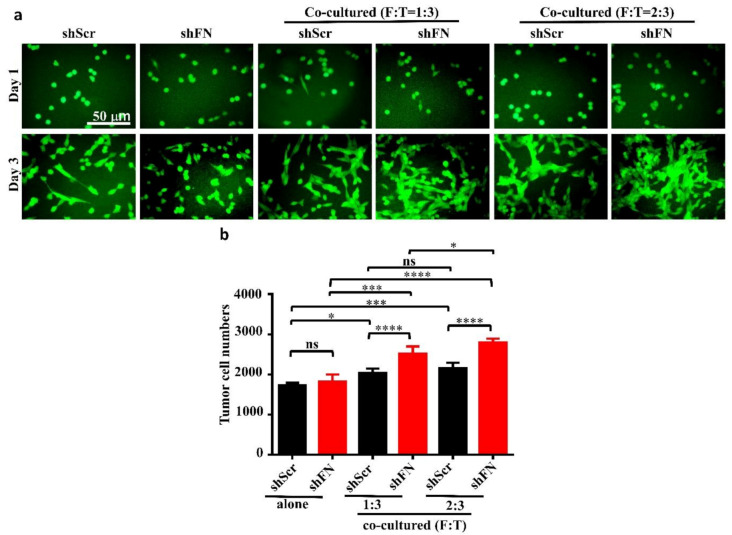
Co-culturing of fibroblasts with periFN-deprived tumor cells facilitates tumor cell proliferation better than with those cells assembling higher level of periFN. (**a**) Representative images for shScr- or shFN-expressing LLC cells either grown alone (two left panels) or co-cultured with fibroblasts at ratio (fibroblasts: LLC cells in cell numbers; F:T) of 1:3 (two middle panels) or 2:3 (two right panels) for 1 (upper panels) or 3 (lower panels) days. (**b**) Quantifications of tumor cell numbers measured by counting numbers of eGFP^+^ tumor cells from images photographed in experiments performed on day 3 in (**a**) with Image J1.x software. Note: all images share the same scale bar as denoted in the image in the upper left panel and all experiments were repeated at least three times. *: *p* < 0.05; ***: *p* < 0.001; and ****: *p* < 0.0001.

**Figure 8:**
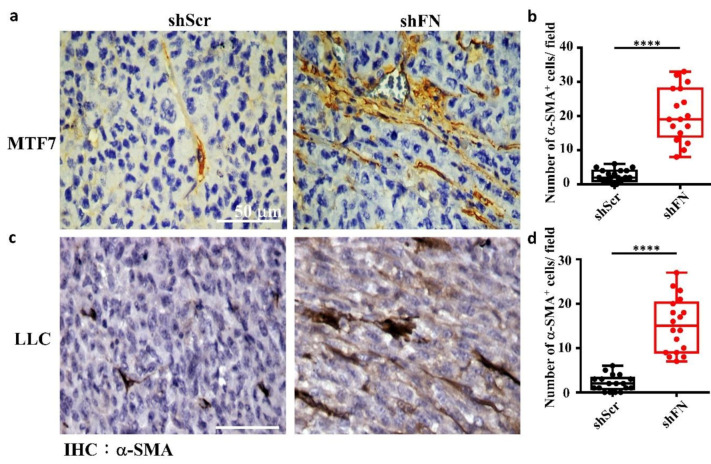
The tumor microenvironments (TMEs) of animals inoculated with shFN tumor cells are infiltrated with more activated fibroblasts than those inoculated with shScr tumor cells. IHC staining with anti-α-SMA antibody for activated fibroblasts existing in TMEs and well aligned with the stretched MTF7 (**a**) or LLC (**c**) cells stably transfected with shScr (left panels) or shFN (right panels) DNA construct. Quantifications for the numbers of α-SMA^+^ fibroblasts per image field (0.015 mm^2^) infiltrated within the TMEs of MTF7 (**b**) or LLC (**d**) tumor tissues. Note: all images share the same scale bar as denoted in the image in the upper left panel and all experiments were repeated at least three times. ****: *p* < 0.0001.

**Figure 9:**
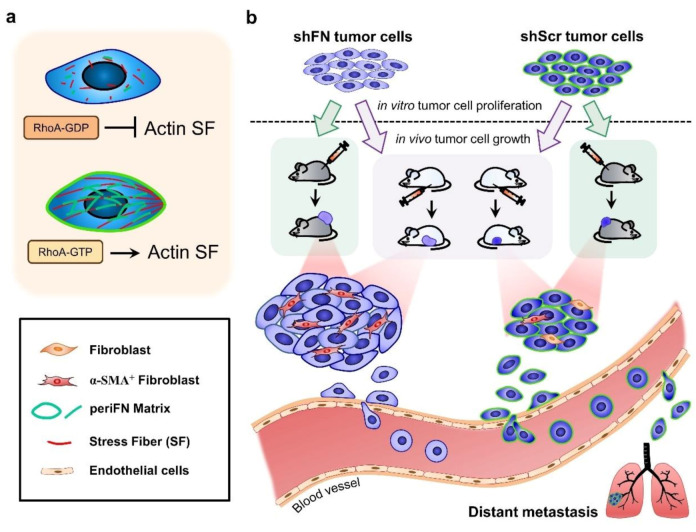
Schematic illustration for the role of tumor-activated fibroblasts within TMEs in promoting in in vivo proliferation of FN matrix-depleted tumor cells. (**a**) The periFN assembly on tumor cells is regulated by RhoA activity-dependent actin SF assembly and alignment. (**b**) Silencing FN expression remarkably suppresses periFN assembly on adherent tumor cells. Although the in vitro tumor cell proliferation is not autonomously affected by FN silencing, the in vivo tumor growth is non-autonomously enhanced by α-SMA^+^ fibroblasts. According to previous findings, however, only tumor cells capable of assembling higher levels of periFN within the circulation are competent in metastasizing to distant organs.

Given that depletion of cancerous periFN matrices did not affect in vitro tumor cell proliferation (Figure 6) but significantly promoted in vivo tumor growth (Figure 5), we continued to test the possibility that TMEs non-autonomously participate in the enhanced in vivo tumor growth in response to cancerous periFN depletion. It has been reported that fibroblasts within TMEs can be activated and driven by tumor cells to facilitate in vivo tumor progression in return [8]. Thus, we hypothesized that tumor cells lacking periFN matrices are more competent than those cells assembling high levels of periFN matrices in activating fibroblasts, consequently enhancing the tumor proliferation within TMEs. Indeed, although both shScr-LLC cells and shFN-LLC cells co-cultured with different ratios of fibroblasts grew better than those cells without being co-cultured with fibroblasts which exhibited similar proliferative activities as shown in Figure 6 (Figure 7a,b), shFN-LLC cells proliferated significantly faster than shScr-LLC cells did once co-culture with fibroblasts in a tumor cell/fibroblast ratio-dependent manner (Figure 7b). To rationalize the role of fibroblasts in the promoted in vivo tumor growth, we performed IHC staining of tumor tissues derived from animals bearing shScr- or shFN-tumor cells for the expression of α-smooth muscle actin (α-SMA), a biomarker for the activated fibroblasts. In line with the results derived from in vitro tumor cell proliferation assays when co-cultured with fibroblasts (Figure 7), we firmly demonstrated that tumor tissues bearing both shFN-MTF7 cells (Figure 8a in right panels and Figure 8b for the corresponding quantification) and shFN-LLC cells (Figure 8c in right panels and Figure 8d for the corresponding quantification) were infiltrated with significantly higher number of α-SMA-positive (α-SMA^+^) fibroblasts that were elongated and aligned well with tumor cells than those tissues bearing shScr-MTF7 cells (Figure 8a in left panels and Figure 8b for the corresponding quantification) and shScr-LLC cells (Figure 8c in left panels and Figure 8d for the corresponding quantification) did. Altogether, we concluded that RhoA-activated SF cytoskeleton enables tumor cells to assemble periFN matrices (Figure 9a) and suppressing such periFN assembly with shFN non-autonomously promote fibroblast-mediated tumor growth (Figure 9b). Interestingly, based on our previous studies [1,9,10,11,12,13], only periFN^+^, but not periFN^-^, tumor cells are competent in distant metastasis once they intravasate and become blood-borne (Figure 9b).

## 3. Discussion

It seems relatively controversial that periFN promotes cancer metastasis but conversely serves as a tumor suppressor to decrease primary tumor cell proliferation [1]. Our findings, to our best knowledge, are the first to reconcile such controversy by showing that depletion of periFN on tumor cell surfaces did not exhibit a direct effect on in vitro tumor cell proliferation but contributed to the driving force toward TMEs that fostered fibroblasts in non-autonomously promoting in vivo tumor growth. Such cross-talk between tumor cells and fibroblasts suggested that the presence of periFN on tumor cells, although pro-metastatic, hampers the recruitment and activation of fibroblasts which play essential roles in promoting tumor progression in the TMEs [8]. Indeed, cancer-associated fibroblasts (CAFs), a distinct population from housekeeping fibroblasts, have often been deemed as a key player throughout tumor progression, including tumor transformation, progression, and even cancer metastasis [45]. Remodeled extracellular matrix (ECM) to which tumor cells attach critically provides pro-tumor cues in TMEs nurturing tumor growth [46]. CAFs, chronically activated fibroblasts by tumor cells in primary tissues, serve as a primary source responsible for ECM component expressions and the subsequent ECM remodeling [8,47]. Interestingly, it has been revealed that various pro-inflammatory factors such as tumor-secreted interleukin-1 and -6 (IL-1 and IL-6) mediate activation of CAFs depending on NF-κB and signal transducer and activator of transcription (STAT) transcription factors, respectively [48,49]. Altogether, these findings denote a possibility that pro-metastatic periFN indirectly participates in non-autonomous inhibition of tumor growth by blockading CAF activation and ECM remodeling that evoke inflammatory responses in TMEs, coordinately promoting primary tumor growth. Nevertheless, it is worth noting that our results did not rule out the possibility of the involvement of other mesenchymal cell types and factors than fibroblasts in promoting primary tumor growth [1].

Now that periFN plays a major role in controlling fibroblast activity, understanding how periFN is regulated in tumor cells may help identify signaling pathways essential for modulating TMEs against tumor growth. Based on our results, endogenous FN expression is required for periFN assembly on tumor cells. TGF-β is one of several well-known players conveying activities of upregulating endogenous FN expression [50,51]. Intriguingly, TGF-β has also been demonstrated to be a tumor suppressor in early tumor stages but promote later distant metastasis [52,53], entertaining a possibility that, whereas TGF-β upregulates FN synthesis, it impedes CAF activation which is one of key factors contributing to tumor growth. In line with this notion, it has been reported that TGF-β potently inhibits secretion of human hepatocyte growth factor (hHGF), a pro-tumor scatter factor with an anti-apoptotic activity, by activated fibroblasts [54,55] and suppresses tumor necrosis factor-induced proliferation of diploid fibroblasts [56] within the pro-tumor growth TMEs. In order to gain early tumor growth advantages, periFN assembly needs to be suppressed, rendering CAF activation to subsequently promote tumor growth until late stages of tumor progression when tumor cells are prepared for distant metastasis [1]. Since periFN assembly is regulated by the tumor suppressor pVHL [34], it is possible that inactivation of pVHL promotes tumor growth due to depletion of periFN, leading to activation of fibroblasts for fostering tumor cells. Consistently, CAFs has currently been reported to be indeed activated in renal cell carcinoma (RCC) with VHL gene malfunction in which RCC cells are deficient in assembling periFN matrices [57]. Unavoidably, once tumor grows up to ~100–200 µm away from blood vessels a hypoxic environment is created [58,59]. To conquer such deleterious conditions, hypoxia-induced factor-1α (HIF-1α) is upregulated in tumor cells to prevent cell apoptosis and remain survived [1]. Indeed, HIF-1α is another intracellular factor in upregulating FN expression [60,61]. Interestingly, HIF-1α is unstable in normaxia conditions due to pVHL-mediated ubiquitylation and degradation of HIF-1α but stabilized in hypoxic conditions where pVHL is inactivated [62]. Whether HIF-1α upregulates FN expression and promotes periFN assembly in the late stage of tumor progression in a pVHL-independent manner warrants further investigation.

Although controversial, the roles of RhoA GTPase and actin SF in tumor growth and metastasis have been extensively studied [37,43,63,64,65]. Abundant evidence indicates that periFN assembly on fibroblasts or endothelial cells is regulated and aligned by RhoA-activated actin SF cytoskeleton [32,33]. However, whether the RhoA/SF axis regulates tumor growth and metastasis through organizing periFN assembly remains uninvestigated. Here, we demonstrated for the first time that in vivo tumor growth could be reduced when the TMEs were devoid of CAFs which could be recruited and activated when RhoA/SF-aligned periFN was absent on tumor cell surfaces. In line with our findings, RhoA has been shown to serve as tumor suppressor in that loss of RhoA activity promotes skin tumor formation [66]. Moreover, actin SF organization is one of the well-known biomarkers for tumor cells experiencing senescence [67,68]**,** indicative of a tumor suppressive role. All this evidence supports the possibility that RhoA/SF-aligned periFN assembly suppresses in vivo tumor growth. Conceivably, depletion of periFN assembly by silencing endogenous FN expression did not affect tumor cell proliferation (Figure 6) as the actin SF cytoskeleton, which critically participates in cell proliferation [4,69,70], was not changed in shFN tumor cells (Figure 3) unless tumor cells were co-cultured with tumor cell-driven fibroblasts which subsequently activated tumor proliferation (Figure 7). Despite the fact that the SF formation was suppressed by RhoA-DN in tumor cells, it is worth investigating the hypothesis that the tumor suppressive role of RhoA [4,66], resulting in unaltered tumor cell proliferation. In congruence with the pro-metastatic property of periFN, RhoA, and actin SF, despite their tumor suppressive roles in the early tumor development, have been shown to endow tumor cells with metastatic-promoting activities in late stages of tumor progression [64,71]. For example, the RhoA/ROCK signaling pathway has been found to participate in IL-6-mediated tumor invasion and distant metastasis [72]. It has been shown that actin SF plays an essential role in promoting migration and metastasis of triple negative breast cancer in which RhoA can be activated by the interaction between Rhophilin-associated tail protein 1 (ROPN-1) and rhophilin-1 to enhance actin SF formation [71]. Conversely, metastasis of esophageal squamous cell carcinoma is suppressed once RhoA is inactivated due to inhibition of phosphorylation of focal adhesion kinase (FAK) [64].

Attempts to target cancerous FN or periFN for cancer treatments and metastatic prevention have been severely hindered due to the paradoxical roles of FN, being tumor suppressive and pro-metastatic [1]. Here, we resolve such a dilemma by showing that depleting periFN on cancer cells, despite preventing blood-borne cancer cells from colonizing distant organs [11,12,13,37], did not decrease cancer cells’ viability and proliferation (Figure 6). Nevertheless, loss of FN expression in cancer cells resulted in in vivo tumor growth, suggesting a role of TMEs in it. Indeed, cancer cell proliferation was enhanced only when co-cultured with fibroblasts, essential mesenchymal cells in TMEs activated in response to stimulus derived from FN-depleted cancer cells (Figure 7). The activated fibroblasts in turn elevated cancer cell proliferation. Therefore, it is an ideal strategy to target cancerous FN for metastatic prevention, whereas therapeutics targeting activated fibroblasts in TMEs may be concomitantly used to prevent tumor growth due to loss of FN expression. Ample evidence indicates that targeting CAFs may turn cancers from foe to friends [45]. In more than 90% of human cancers, fibroblast activation protein (FAP) is highly expressed in activated stromal fibroblasts, responsible for myofibroblast recruitment, differentiation, and proliferation [73]. Preclinically, PT630 (GluBoroPro dipeptide), a pharmacological inhibitor of FAP, has been well demonstrated to cause a drastically reduced infiltration of myofibroblasts into tumor tissues, resulting in diminished tumor growth [74]. Vaccination against FAP expressed on CAF surfaces has also been attempted for controlling tumor growth by directly depleting the activated CAFs [75]. Reverting the activated CAFs into quiescence has been a replacement approach in cancer therapies [45]. For instance, administration of all-trans retinoic acid to enrich vitamin A or calcipotriol, a ligand of vitamin D receptor, to facilitate vitamin D functions have been shown to reset the activated fibroblasts to an inactive state [76,77]. Moreover, direct depleting or inactivating CAFs in primary tumor tissues may contribute to metastatic suppression by preventing them from creating pre-metastatic niches in distant organs [78,79,80]. Whether simultaneously targeting periFN assembled on cancer cells and CAFs in TMEs in combinatory cancer therapies concomitantly impede distant cancer metastasis and prevent primary and secondary tumor growths merits further investigation.

In addition to CAF/periFN-dual targeting cancer combination therapies, alternative strategies may be considered. The phytochemical pterostilbene (PS), a resveratrol derivative, has been identified to exhibit dual roles in combating cancer metastasis [12]. In an animal cancer metastasis model, it has been shown that orally administered PS potently inhibited distant metastasis of suspended tumor cells in the circulation through suppressing cancerous periFN assembly due to AKT activation followed by ERK inactivation in an apoptosis-independent manner, whereas it drastically caused apoptosis of adherent tumor cells via AKT inactivation in solid tumor tissues [12]. Such dual roles of PS directly render apoptosis of tumor cells resided either in primary tissues or in distant organs regardless of the likely stimulatory effect of PS on tumor growth in solid tissues due to the reduced periFN assembly on tumor cells. The fact that AKT activity can be differentially regulated in adherent and suspended cancer cells leads to a possibility that periFN assembly can also be oppositely regulated. Thus, it is worthwhile in the future to explore another possible combination therapeutic strategy in which drugs possess a dual role in concomitantly inhibiting periFN assembly on suspended tumor cells and promoting that on adherent tumor cells.

## 4. Materials and Methods

### 4.1. Cell Lines

Lewis lung carcinoma (LLC) cell line (ATCC: CRL-1642, Richmond, VA, USA) was purchased from the American Type Culture Collection. MTF7 cell line cloned from rat mammary adenocarcinoma 13762NF cells was received as a kind gift from D. R. Welch at Pennsylvania State College of Medicine, Hershey, PA. Normal rat kidney interstitial fibroblast (NRK-49F) cell line was obtained from M. J. Tang at National Cheng Kung University College of Medicine. These cell lines were all cultured in Dulbecco’s modified Eagle’s medium (DMEM) containing 2 mM L-glutamine and supplemented with 8% fetal bovine serum (FBS) (Gibco BRL, Waltham, MA, USA). MTF7 cell line was cultured in the same culture medium but containing additional 1 mM sodium pyruvate (Sigma G6013, St. Louis, MO, USA). Tissue culture plasticware was either purchased from BD Falcon (Franklin Lakes, NJ, USA) or from Wuxi NEST. Biotechnology Co., Ltd. (Wuxi, Jiangsu, China).

### 4.2. Materials

All pharmaceutical compounds unless otherwise indicated and polyclonal antibodies (pAbs) against FN were purchased from Sigma-Aldrich, Inc. (St. Louis, MO, USA). 4′,6-diamidino-2-phenylindole (DAPI), Hoechst 33258, Phalloidin-Alexa 594 (A-12381), goat anti rabbit (GαR)-Alexa 488 (A11008), GαR-Alexa 594 (A11035) and pcDNA™3.1/pcDNA-eGFP were from Invitrogen (Waltham, MA, USA). Rabbit pAb against α-smooth muscle actin (α-SMA) was from ProteinTech Group, Inc. (Chicago, IL, USA). Control rabbit non-immune IgG was from Jackson Immunoresearch Laboratories, INC (West Grove, PA, USA). The RNAi reagents for lentiviral vector system were from the National RNAi Core Facility supported by the National Research Program for Genomic Medicine Grants of NSC (NSC 100-2314-B-006-055). Bovine serum albumin (BSA), CyECL reagents and Cytochalasin D, an inhibitor of actin polymerization, were from Cyrusbioscience (Taipei, Taiwan). Annexin V-FAM apoptosis detection reagent was from LEADGENE (Tainan, Taiwan). TAlink mouse/rabbit polymer detection system (TAHC04D) was from BioTnA (Kaohsiung, Taiwan). Immobilon-P Poly vinylidene fluoride (PVDF) Membrane (IPVH00010) was from Sigma-Aldrich; Merck Millipore (Darmstadt, Germany). 12 mm microscope cover glasses were from Glaswarenfavrik Karl Hecht GmbH and Co. (Sondheim, Germany). Plasmids pRK5, p-RK5-RhoA-DN (RhoA-DN), and p-RK5-RhoA-CA (RhoA-CA) [81] were kindly received from Dr. A. Hall at MRC Laboratory for Molecular Cell Biology and Cell Biology Unit, London, UK.

### 4.3. Silencing Endogenous FN Expression in Tumor Cells with Short Hairpin RNAs Complementary to FN mRNA

The lentiviruses for RNAi techniques were produced as previously described [82]. LLC and MTF7 cells infection with lentiviruses carrying shScr or shFN and selection for stable cells with puromycin (5 μg/mL for LLC cells and 7 μg/mL for MTF7 cells) were performed as previously described [12].

### 4.4. Immunoblotting

Total MTF7 or LLC cell lysates were prepared and subjected to SDS-PAGE electrophoresis, electrotransferration, and western immunoblotting (IB) as previously described [9]. The chemiluminescence images for IB on PVDF membranes were developed with CyECL reagents according to the manufacturer’s instruction. Internal loading controls were performed with Coomassie Blue staining of the same blotted membranes.

### 4.5. Immunofluorescence Staining for periFN Matrices on Adherent Tumor Cell Surfaces

LLC cells (1.8 × 10^5^) or MTF7 (7.5 × 10^4^) cells were seeded on 12 mm sterilized microscope cover glasses placed in 3 cm culture dishes containing growth media supplemented with 8% FBS for 48 h. Cells were fixed with 1% paraformaldehyde (PFA) in phosphate-buffered saline (PBS), pH 7.4 at 37 °C for 10 min. Before immunofluorescence (IF) staining, the cover glasses were removed from 3 cm culture dishes and placed into 24-well dishes. After three times of washes, cells were incubated for 1 h at 37 °C with aFN pAb or non-immune control IgG followed by 1 h incubation of GαR Alexa 488 (in green) or 594 (in red) antibodies and DAPI mounting for nuclear staining (in blue). The cover glasses were washed again and mounted in 4′,6-diamidino-2-phenylindole (DAPI) on microscope slides and examined and photographed for fluorescence under fluorescence microscope.

### 4.6. Fluorescence Staining for Actin Cytoskeleton

Tumor cells grown on microscope cover glasses, as previously described, were fixed with cytoskeleton staining buffer with sucrose (10 mM MES pH6.1, 138 mM KCl, 3 mM MgCl_2_, 2 mM EGTA, sucrose 0.32 M) [83] for 20 min and permeabilized with 0.5% Triton X-100 at room temperature (RT) for 3 hr. The cells were then blocked with 0.1% Triton X-100 and 2% BSA in PBS at RT for 10 min and labeled with Phalloidin-Alexa 594 (1/500 dilution from the stock solution) at 37 °C for 20 min prior to DAPI mounting for nuclear staining. Cells were washed at least three times with PBS after each step during the staining procedure. For Cytochalasin D (CD) treatments, cells were pretreated with 0.1 or 0.5 µM of CD at 37 °C for 12 h before Phalloidin staining.

### 4.7. Double Fluorescence Staining for periFN and Actin Stress Fiber in Tumor Cells

Tumor cells without permeabilization were first subjected to IF staining (in green) prior to cell permeabilization and actin cytoskeleton fluorescence staining (in red) as previously described. DAPI was used for nuclear staining (in blue).

### 4.8. Plasmid Transfection of Tumor Cells by Electroporation

The transfection protocols were performed using Neon^TM^ Transfection System (MPK5000; Invitrogen, Carlsbad, CA, USA) according to manufactural instruction. Briefly, MTF7 cells or LLC cells (1 × 10^6^) resuspended in 100 µL of resuspension buffer R containing 4 µg of DNA plasmids (vector, RhoA-DN, RhoA-CA, or pcDNA-eGFP) were uptaken into the Neon Tip connected to the top-head of Neon Pipette that was subsequently placed in the Neon Tube containing 3 mL of electrolytic buffer E before the entire assembly was set up in the Neon Pipette Station. Cells were then electroporated with 1230 V, 40 ms, 1 pulse for MTF7 cells, or with 1200 V 20 ms 1 pulse for LLC cells at RT. The electroporated cells were immediately reseeded in culture dishes for 48 h before being subjected to PFA fixation and fluorescence staining. LLC and MTF7 cells carrying pRK5 plasmid alone or expressing RhoA-DN or RhoA-CA that were co-transfected with pcDNA with Neo gene and LLC cells expressing pcDNA-eGFP alone were selected with G418 (neomycin) antibiotics to establish stable cell lines respectively expressing exogenous proteins.

### 4.9. Real-Time Cell Proliferation and Co-Culture Assays

Tumor cells (2 × 10^4^) were first seeded in 24-well dishes for 24 h and subjected to fluorescent microscopic imaging every 24 h upon UV-excitation after 10-min treatment with 5 µg/mL of Hoechst 33258 dye that was used to nuclear staining in live cells. At the time point of each experiment, Hoechst 33258 dye-positive cells were imaged and counted with Image J software. For co-culture assays, 3 × 10^4^ of LLC-shScr or -shFN cells stably expressing GFP were either seeded alone or co-seeded with 1 × 10^4^ (NRK-49F: LLC = 1:3) or 2 × 10^4^ (NRK-49F: LLC = 2:3) of rat NRK-49F fibroblasts and subjected to fluorescent (eGFP for tumor cell counting) microscopic imaging for the same time intervals as abovedescribed. Numbers of eGFP^+^ tumor cells were counted with Image J software.

### 4.10. Apoptosis Assay

The apoptosis assays employing propidium iodine (PI)/Annexin V-FAM double fluorescence staining were performed as previously described [12]. Briefly, tumor cells were cultured for 48 h prior to double fluorescence staining. The stained tumor cells were subjected to fluorescence detection by flow cytometry and FACS analysis. The percentage of annexin V^+^ and PI^+^ cells were calculated.

### 4.11. In Vivo Tumor Growth Animal Models

All animal experiments were performed according to the Guide for Care and Use of Laboratory Animals at National Cheng Kung University (NCKU) and experimental protocols were approved by NCKU Internal Laboratory Animal Care and Use Committee (IACUC) at School of Laboratory Animal Center. C57BL6 mice and F344 rats were acquired from the School of Laboratory Animal Center at NCKU and housed by five per cage at 24 ± 2 °C and 50 ± 10% relative humidity, which were subjected to a 12-h light/12-h dark cycle. For shFN effects in vivo tumor growth experiment, C57BL6 mice subcutaneously bearing 1 × 10^5^ shScr-LLC or shFN-LLC cells or F344 rats bearing 6 × 10^5^ shScr-MTF7 or shFN-MTF7 cells in mammary fat pads waited for 20 days prior to animal sacrifices by CO_2_ euthanasia. Tumor volumes were measured.

### 4.12. Immunohistochemistry Staining

IHC staining for activated fibroblasts in TMEs of animals inoculated with MTF7 or LLC cells was performed as previously described [84]. Briefly, slides of paraffin-embedded and formalin-fixed tumor tissues were first deparaffinized and rehydrated with graded xylene/ethanol solution followed by antigen retrieval with Tris-EDTA (pH 9.0) under high pressure of 1.5 kg/cm^2^ at 120 °C for 20 min. The retrieved tissues were then subjected to TAlink mouse/rabbit polymer detection system according to manufactural instruction.

### 4.13. Statistical Analysis

Statistical analyses were performed using GraphPad Prim6. Student’s t-test were performed to compare paired data, One-Way ANOVA compare the means of two or more independent groups, and Two-Way ANOVA analyze variance and test differences in the effects of independent variables on a dependent variable like tumor cell proliferation. All experiments were at least independently performed in biological triplicate, and the results are shown as mean ± S.D. The differences were considered as the *p* value which *p* < 0.05 (*), *p* < 0.01 (**), *p* < 0.001 (***), *p* < 0.0001 (****).

## Abbreviations

FN	fibronectin
periFN	pericellular FN
SF	stress fiber
TMEs	tumor microenvironments
VHL	von Hippel–Lindau
RhoA-DN	dominant-negative Thr19Asn RhoA
GEF	guanidine nucleotide exchanging factor
RhoA-CA	constitutively active Gln63Leu RhoA
CAFs	cancer associated fibroblasts
α-SMA	α-smooth muscle actin
ECM	extracellular matrix
IL-1	interleukin-1
IL-6	interleukin-6
hHGF	human hepatocyte growth factor
HIF-1α	hypoxia-induced factor-1α
ROPN-1	Rhophilin-associated tail protein 1
FAP	fibroblast activation protein
